# The reproducibility of dendritic cell and T cell counts to a 30‐min high‐intensity cycling protocol as a tool to highlight overtraining

**DOI:** 10.1113/EP091326

**Published:** 2023-12-08

**Authors:** Carla Baker, Jessica Piasecki, John A. Hunt, John Hough

**Affiliations:** ^1^ Department of Sport Science SHAPE Research Centre, Nottingham Trent University Nottingham UK; ^2^ Medical Technologies Innovation Facility Nottingham Trent University Nottingham UK

**Keywords:** dendritic cells, immune biomarkers, intensified exercise, reproducibility, T cells

## Abstract

Heavy training has been reported to be immunosuppressive in athletes and lead to blunted cortisol responses to exercise. Cortisol elevates the number of dendritic cells (DCs), key antigen‐presenting cells that interact with T cells to initiate an immune response. Reproducible cortisol responses to a 30‐min cycle test have been identified but were based on percentage of work rate maximum. To ensure physiological consistency, submaximal anchors, that is, ventilatory threshold (VT_1_) should prescribe intensity. This study aims to assess the reproducibility of the DC and T cell responses to an adapted stress test to assess its usefulness in assessing DC dysfunction with intensified training. Twelve males cycled for 1 min at 20% below VT_1_ and 4 min at 50% between VT_1_ and ${\dot{V}}_{O_{2}\max}$, for 30 min (20/50), with blood samples pre‐, post‐ and 30 min post‐exercise. This was repeated twice, 2–7 days apart. Flow cytometry assessed total DCs, plasmacytoid DCs, myeloid DCs, total T cells, T helper cells and T cytotoxic cells. No significant trial or interaction effects were found for any variable. A significant main effect of time for all variables was found; immune cells increased from pre‐ to post‐exercise and decreased to baseline 30 min post‐exercise, apart from plasmacytoid DCs, which remained elevated 30 min post‐exercise. Intraclass correlation coefficients showed overall good‐to‐excellent reliability for all immune cells, with smallest real difference and Bland–Altman analysis verifying high reproducibility between trials. These results suggest that the 20/50 exercise test induces reproducible DC and T cell count changes, which, implemented before and after a period of intensified training, may highlight the negative states of overtraining.

## Abstract (highlights)

- **What is the central question of this study?**
  Can we develop an exercise stress test robust enough to elicit reproducible changes in immune cell counts, so that when used before and after a period of intensified training we can examine whether immune changes are useful biomarkers in highlighting the negative states of overtraining?
- **What is the main finding and its importance?**
  An exercise stress test was developed that elicited reproducible and robust changes in dendritic cell and T cell counts, making it a valuable tool in the examination of immune cell counts as possible biomarkers in highlighting the negative states of overtraining.

## 1 INTRODUCTION

As sport continues to push the physiological boundaries of performance, there is a growing expectation for athletes to undertake higher training loads. It is established that successful training regimes involve the fine‐tuned balancing of overloading the body whilst also ensuring adequate recovery (Meeusen et al., 2010). If sufficient recovery is not achieved, short‐term decrements in performance may arise in as little as 7 days (Halson et al., 2002). However, if appropriate recovery is implemented at this point, a ‘supercompensatory’ response in performance can occur, known as functional overreaching (FOR) (Birrer et al., 2013). However, if adequate recovery is not implemented alongside the high training loads, the athlete may become non‐functionally overreached (NFOR) which, if left undiagnosed, may result in chronic maladaptation that can lead to the overtraining syndrome (OTS) (Schwellnus et al., 2016). Symptoms of NFOR/OTS most commonly occur in endurance events such as swimming, cycling or running (Cardoos, 2015), including both individual (37%) and team (17%) sport athletes (Matos et al., 2011), likely due to the high training volumes required for peak performance. Despite the incidence of NFOR/OTS across an athlete's career spanning as much as 30–60% (Birrer et al., 2013; Morgan et al., 1987), little progress has been made on uncovering objective and reliable biomarkers that focus on identifying the occurrence of NFOR/OTS and the underlying mechanisms leading to the associated illness symptoms (Armstrong & Vanheest, 2002; Schwellnus et al., 2016).

It seems that periods of heavy training, most notably those that induce NFOR/OTS, decrease the immune response in athletes and lead to increased episodes of upper respiratory tract infections (Spence et al., 2007; Walsh, 2019). It has been argued that a cortisol‐driven reduction in circulating cytotoxic T cells (Steensberg et al., 2001) along with a reduction in immunosurveillance post‐exercise stress introduces a ‘window of opportunity’ for infection (Simpson et al., 2020). Dendritic cells (DCs) are key antigen presenting cells involved in linking the innate and adaptive immune systems (Martin‐Gayo & Yu, 2019). Two major subsets of DCs are found in peripheral blood: myeloid DCs and plasmacytoid DCs (pDCs). Myeloid DCs mainly stimulate antigen‐specific T cells, whereas pDCs produce high levels of type 1 interferons and play an important role in anti‐viral defence (Suchanek et al., 2010). Despite their important role in orchestrating the immune response, and their potential as a biomarker in the identification of NFOR/OTS, they have not been readily researched.

Cortisol is a hormone synthesized and secreted by the hypothalamic–pituitary–adrenal (HPA) axis in response to physical and mental stress (Hill et al., 2008). In the immune system cortisol plays an important anti‐inflammatory role in response to exercise by supressing pro‐inflammatory mediators such as reactive oxygen species (Franchimont, 2004), inducing lymphocytopenia (Okutsu et al., 2005) and regulating the maturation and migration of DCs towards lymph nodes (Liberman et al., 2018). During periods of intensified exercise, a maladaptation of the HPA axis has been reported, specifically a blunted cortisol response to exercise (Meeusen et al., 2004). Therefore, a dysfunctional HPA axis may in part lead to impaired immune regulation during intensified training.

This maladaptation in cortisol is not apparent at rest but is highlighted when examining the HPA axis response to exercise stress. Hough et al. (2021, 2011) identified robust and reproducible cortisol increases to a 30‐min high intensity exercise stress test known as the 55/80 in healthy male athletes. This test consists of alternating blocks of 1 min cycling at 55% maximum power output ($\dot{W}$_max_) and 4 min cycling at 80% $\dot{W}$_max_. The same group found a 72% blunting in the cortisol response to the 55/80 test after an 11‐day intensified training period when compared with a test before the intensified training period (Hough et al., 2013). Similarly, Meeusen et al. (2004) used two maximal cycle tests separated by 4 h in well‐trained athletes to show that the exercise‐induced responses of cortisol and adrenocorticotropic hormone (ACTH; a precursor hormone to cortisol) to the second maximal cycle reduced cortisol and ACTH by ∼118% and ∼73%, respectively, after a 10‐day training period compared to before (Meeusen et al., 2004).

However, the 55/80 stress test is based solely on the percentage of work rate maximum, identified from work rate achieved at ${\dot{V}}_{O_{2}\max}$. It has been argued that using a percentage of maximum to prescribe exercise intensity assumes that all participants will experience the same homeostatic perturbations to the same relative intensity, not taking into account submaximal physiological thresholds (Jaminick et al., 2020). However, large differences in homeostatic perturbations, that is, oxygen uptake kinetics and blood lactate responses, have been reported across multiple studies using exercise within the ‘moderate intensity’ zone (60–80% ${\dot{V}}_{O_{2}\max}$) (Jamnick et al., 2020). As such, the use of submaximal anchors, such as the ventilatory threshold, is recommended to prescribe exercise intensity (Mann et al., 2013). Therefore, to ensure the stress test is stressful enough to highlight any immune alterations that may occur due to excessive exercise in all participants, an adjusted version of this 55/80 stress test that prescribes intensity based on the ventilatory threshold (VT_1_) has been developed for use in the current study.

It has been shown that the total number of DCs can be elevated and phenotypic changes induced by 20–30 min of exercise (Brown et al., 2018; Deckx et al., 2015). Additionally, the 30‐min cycling 55/80 stress test used by Hough et al. (2011, 2021) to show reproducible cortisol increases induced a mean heart rate of ∼162 bpm, and a rating of perceived exertion (RPE) of ∼13, which is similar to the mean heart rate of ∼152 bpm and mean RPE of ∼13 elicited by the 20/50 in the current study. These studies suggest that the 30‐min adapted version of the 55/80 stress test (Hough et al., 2011, 2021), the 20/50, may also be stressful enough to elicit robust and reproducible changes in immune cell number. If reproducible DC and T cell count responses also occur in response to the exercise stress test, when implemented before and after a period of intensified training, changes in immune cell count and/or function could be identified as possible biomarkers of NFOR/OTS.

The aim of the current study was to establish the reproducibility of immune cell count responses to an adapted version of the 55/80 stress test developed by Hough et al. (2011) that has previously been shown to elicit reproducible cortisol changes. Specifically, the reproducibility of peripheral blood total DCs, myeloid and pDCs, total T cells and CD8^+^ and CD4^+^ T cells was examined. It was hypothesized that the 30‐min developed exercise stress test, the 20/50, would induce robust and reproducible immune cell count changes, increasing post exercise from baseline and declining back towards baseline 30 min post exercise.

## 2 METHODS

### 2.1 Ethical approval

Prior to study participation, written informed consent was obtained from all participants, and health questionnaires were completed. The study conformed to the standards set by the *Declaration of Helsinki*, except for registration in a database, and procedures were approved by the NTU ethics committee (Ethical approval number 698; Nottingham Trent University, UK).

### 2.2 Participants

Twelve healthy males were included in the study (age: 26.4 ± 5.8 years; height: 182.5 ± 5.3 cm; body mass: 81.6 ± 8.3 kg; body mass index, 24.48 ± 1.98 kg/m^2^; ${\dot{V}}_{O_{2}\max}$, 48.58 ± 7.14 ml/kg/min). All were free from upper‐respiratory tract infections for at least 2 weeks prior to testing and none were taking any medications. All participants were required to undertake structured exercise at least three times per week to be included.

### 2.3 Pre‐experimental procedures

Height (Seca 217 stadiometer, Seca, Hamburg, Germany) and body mass (Seca 761 scales) were collected using standard methods, and cardiopulmonary fitness (${\dot{V}}_{O_{2}\max}$) was assessed on a Lode Excalibur Sport electronically braked cycle ergometer (Lode, Groningen, the Netherlands), using a continuous ramp protocol, starting at 0 W increasing by 30–45 W per minute until volitional fatigue. Expired air was assessed throughout the test for oxygen consumption and carbon dioxide production using breath‐by‐breath analysis (Version 3B, Cortex Biophysik, Leipzig) for ventilatory threshold 1 (VT_1_) and ${\dot{V}}_{O_{2}\mathrm{peak}}$ to be calculated. Heart rate was assessed using a polar heart rate monitor (Polar F2, Polar Electro Oy, Kempele, Finland) and ratings of perceived exertion recorded using the Borg scale (Borg, 1982).

### 2.4 Determination of exercise intensity

The 30‐min cycle (20/50) was split into six blocks of 1 min at 20% below VT_1_, and 4 min at 50% of the difference between power at VT_1_ and ${\dot{V}}_{O_{2}\mathrm{peak}}$. The ${\dot{V}}_{O_{2}\mathrm{peak}}$ was defined as the mean of the highest exertional oxygen uptake achieved over the last 30 s of exercise. The VT_1_ was determined using the modified V‐slope method, confirmed by patterns of change in ventilatory equivalent and end‐tidal gas measurements and verified by an independent researcher. To account for the ramp, power outputs at VT_1_ and ${\dot{V}}_{O_{2}\max}$ were adjusted by subtracting two‐thirds of the ramp increment (i.e., power output – 0.6 × ramp increment).

### 2.5 Exercise trial and blood sampling

**FIGURE 1:**
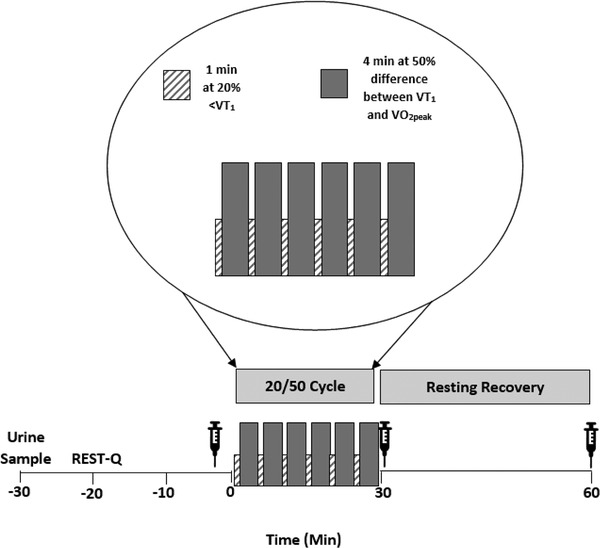
Schematic representation of the 20/50 exercise trials. The syringe symbol represents a venepuncture blood sample.

At least 7 days after participants completed their ${\dot{V}}_{O_{2}\mathrm{peak}}$ test, participants reported to the laboratory for their first exercise trial (Figure 1). Participants completed both exercise trials at the same time of day to ensure limited influence of circadian rhythms (commencement ranging from 09.20 to 13.30 h), 2–7 days apart and were instructed to consume the same foods and to drink at least 500 mL of water on the morning before each visit to ensure hydration. Once in the laboratory, urine osmolality was assessed, with an osmolality of <700 mOsmol/kg being acceptable for blood sampling. If participants did not meet these criteria, they were instructed to consume 500 mL of water, and wait for 10 more minutes before it was repeated. Participants then undertook seated‐rest whilst completing the 76 item Recovery‐Stress Questionnaire for Athletes (RESTQ‐76 Sport) (Kellmann & Kallus, 2001). After ∼15‐min seated‐rest, 8–12 mL blood was collected via venepuncture from the forearm into two 4–6 mL EDTA vacutainers. After a 3‐min warm up at 50 W, the 30‐min 20/50 began, with heart rate and RPE measured 30 s before the end of each interval. Immediately after the cycle, a venepuncture blood sample was collected. Participants rested, then 30 min later the final blood sample was collected.

### 2.6 Peripheral blood mononuclear cell isolation and flow cytometry

Four millilitres of EDTA‐treated whole blood was layered onto 4 mL of Ficoll‐Paque PLUS (GE Healthcare, Chicago, IL, USA) and centrifuged at 400 *g* for 25 min at 20°C. Peripheral blood mononuclear cell (PBMCs) were harvested and washed in phosphate buffered saline (PBS) by centrifuging at 400 *g* for 5 min. Cells were resuspended in 4 mL PBS and counted using a haemocytometer before being split into two 2 mL suspensions, washed in PBS for 7 min at 500 *g* and resuspended in 100 μL PBS. Fluorescently conjugated antibodies were added to approximately 1 × 10^6^ cells before a 30‐min incubation in the dark at room temperate to identify DC and T cell subpopulations. The following monoclonal antibodies were used: FITC‐conjugated anti‐Lineage 2 cocktail (CD3 clone no. SK7, CD19 clone no. SJ25C1, CD20 clone no. L27, CD14 clone no. MφP9, CD56 clone no. NCAM16.2), PerCP‐Cy 5.5‐HLA‐DR‐conjugated clone no. G46‐6, APC‐conjugated CD11c clone no. S‐HCL‐3, PE‐conjugated IL‐3Rα (CD123) clone no. 6H6, FITC‐conjugated CD3 clone no. HIT3a, PE‐conjugated CD4 clone no. RPA‐T4, and APC‐conjugated CD8 clone no. RPA‐T8 (BD Biosciences, San Diego, CA, USA). After incubation, the PBMCs were washed in PBS for 7 min at 500 *g* and resuspended in 200 μL PBS. Samples were run for 30,000 events in four‐colour flow cytometry (Accuri C6, BD Biosciences).

### 2.7 Flow cytometry analysis

Data were analysed using the Kaluza Analysis software (Beckman Coulter, Brea, CA, USA). PBMCs were gated on the forward versus side‐scatter. Total DCs were identified as being lineage‐HLA‐DR^+^, and further analysed for CD11c and CD123 to identify plasmacytoid DCs (CD11c^−^ CD123^+^) and myeloid DCs (CD11c^+^ CD123^−^). Total T cells were identified as being CD3^+^ and were further analysed for CD4^+^ and CD8^+^ to identify T helper (CD3^+^ CD4^+^ CD8^−^) and T cytotoxic cells (CD3^+^ CD4^−^ CD8^+^).

### 2.8 Statistical analysis

Data were examined using SPSS Statistics version 28 (IBM Corp., Armonk, NY, USA) for normal distribution using the Kolmogorov–Smirnov test. Non‐normally distributed data were logarithmically transformed and re‐examined. Data that were still non‐normally distributed were analysed using non‐parametric statistical tests. Wilcoxon's signed‐rank test was used to assess main effect of trial and *post hoc* time effects, and a Friedman's test was used to assess main effects of time. This was performed for plasmacytoid DCs only. Normally distributed immune cell count responses to the 20/50 exercise test were assessed using a two‐way repeated measures analyses of variance (ANOVA) to examine the effects of trial (trials 1 and 2) and time on the immune cell counts between 20/50 trials. When the assumption of sphericity was violated, a Greenhouse–Geisser correction was applied. Student's paired samples *t*‐test was used to examine differences between HR, RPE and REST‐Q questionnaire scores between trials. Statistical significance was accepted at the *P* < 0.05 level. Data are presented as means ± standard deviation (SD).

Relative reliability between the two 20/50 trials was assessed by calculating the intraclass correlation coefficients (ICC) and the intra‐individual coefficient of variation (CV). ICC was calculated via the ICC_2,1_ model for the post‐exercise cell counts using IBM SPSS Statistics version 28. ICC values <0.40 indicate poor reliability, 0.40−0.59 indicate fair reliability, 0.60−0.74 good reliability and >0.75 indicates excellent reliability (Fleiss, 1981). CV (%) was calculated from intra‐individual delta pre to peak post cell counts and is presented alongside 95% confidence intervals (Table 2). Absolute reliability was assessed by Bland–Altman plot analysis and limits of agreements, and calculation of the closely related smallest real difference (SRD) was performed as described in Madsen et al. (2023) (Table 2).

## 3 RESULTS

### 3.1 Physiological and recovery‐stress data: HR, RPE and REST‐Q questionnaire

#### 3.1.1 Heart rate

Paired samples *t*‐tests showed no significant difference in mean heart rate between the two 20/50 exercise trials (*t*(11) = 0.610, *P* = 0.554) (Table 1 ).

**TABLE 1:** Mean ± SD of heart rate (bpm) (*n* = 12), rating of perceived exertion (*n* = 12) and REST‐Q (*n* = 11) mean total stress and recovery scores for the 20/50 trial 1 and trial 2.

	20/50 trial 1	20/50 trial 2	*P*
Heart rate (bpm)	153 ± 14	152 ± 15	0.554
RPE	14 ± 1	13 ± 1	0.086
REST‐Q			
Total stress	1.7 ± 0.5	1.6 ± 0.5	0.442
Total recovery	3.0 ± 1.1	2.9 ± 1.1	0.476

Paired samples *t*‐tests were used to assess significance. RPE, rating of perceived exertion.

**TABLE 2:** Mean intra‐individual coefficients of variation (%) with 95% confidence intervals for pre‐, post‐ and 30 min post‐20/50, and the smallest real difference (SRD) for delta pre‐ to peak post‐exercise cell counts (*n* = 12).

	CD3^+^ T cells	CD4^+^ T cells	CD8^+^ T cells	Total DCs	mDCs	pDCs
Coefficient of variation (%) (95% CI)						
Pre‐20/50	12.9 (9.8, 16.0)	13.6 (10.0, 17.2)	12.8 (9.9, 15.7)	19.1 (10.0, 28.2)	21.2 (11.02, 31.3)	14.0 (5.7, 22.2)
Post‐20/50	7.7 (4.0, 11.4)	8.9 (5.5, 12.3)	8.9 (4.7, 13.1)	15.1 (7.4, 22.8)	18.1 (8.0, 28.3)	15.3 (8.3, 22.2)
30 min post‐20/50	14.1 (8.3, 19.8)	13.9 (8.3, 19.4)	14.5 (8.8, 20.2)	25.2 (14.3, 36.2)	31.5 (19.0, 44.1)	23.43 (15.5, 31.4)
SRD (95% CI)						
	2.7 (2.2, 3.2)	0.7 (0.6, 0.9)	1.3 (1.1, 1.6)	1.8 (1.5, 2.1)	1.5 (1.2, 1.7)	0.2 (0.2, 0.3)

#### 3.1.2 RPE

Paired samples *t*‐tests showed no significant difference in RPE between the trial 20/50 exercise trials (*t*(11) = 1.887, *P* = 0.086) (Table 1).

#### 3.1.3 REST‐Q

Paired samples *t*‐tests showed no significant differences in the mean of total stress (*t*(10) = 0.800, *P* = 0.442) or the mean of total recovery (*t*(10) = 0.740, *P* = 0.476) (Table 1).

### 3.2 T cells

#### 3.2.1 Total T cells (CD3^+^)

**FIGURE 2:**
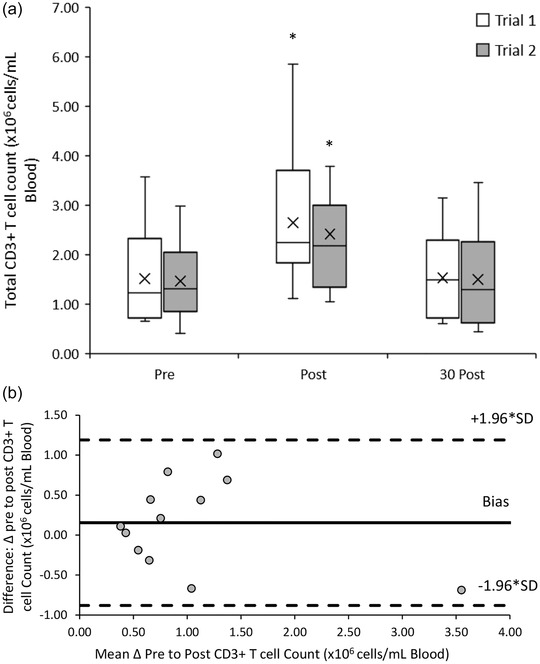
(a) CD3^+^ T cell counts pre‐, post‐ and 30 min post‐20/50 exercise bouts in both repeated trials. Counts are presented as means ± SD × 10^6^ cells/ml blood. *Means significantly different from baseline (pre‐exercise), *n* = 12. (b) Bland–Altman plot: differences between delta (Δ) pre to post CD3^+^ T cell counts between trial 1 and 2 by the mean of the two. Dashed lines indicate the upper and lower limits of the 95% confidence interval for the mean differences, *n* = 12.

The CD3^+^ T cell response to both 20/50 exercise trials was similar (*F*(1, 11) = 1.191, *P =* 0.299). There was a significant main effect of time (*F*(2, 22) = 39.237, *P* < 0.001) with acute increases in peripheral blood CD3^+^ from pre‐ ((1.49 ± 0.82) × 10^6^ cells/ml) to post‐exercise ((2.53 ± 1.34) × 10^6^ cells/ml; *P* < 0.001), followed by a decrease towards baseline at 30 min post‐exercise ((1.51 ± 0.89) × 10^6^ cells/ml; *P* < 0.001). There was no significant interaction effect between trial and time point (*F*(2, 22) = 0.524, *P* = 0.600) (Figure 2a).

An excellent reliability in the responses of CD3^+^ T cell counts to the 20/50 exercise trials was found with an ICC of 0.970 (95% CI: 0.891, 0.992). Bland–Altman analysis indicates that the differences in CD3^+^ T cell counts between trials ranged from 1.02 to −0.69 × 10^6^ cells/ml blood. One hundred percent of CD3^+^ T cell measurements lay within the upper (1.19 × 10^6^ cells/ml blood) and lower (−0.88 × 10^6^ cells/ml blood) limits of agreement (Figure 2b).

#### 3.2.2 CD4^+^ T Cells

**FIGURE 3:**
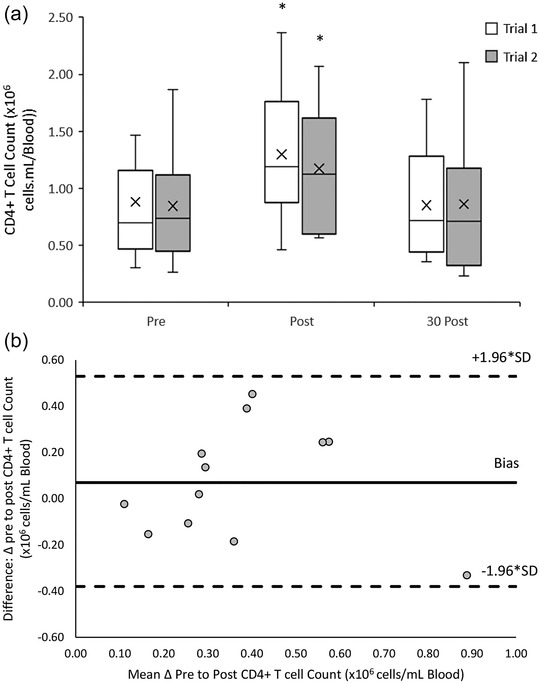
(a) CD4^+^ T cell counts pre‐, post‐ and 30 min post‐20/50 exercise bouts in both repeated trials. Counts are presented as means ± SD × 10^6^ cells/ml blood. *Means significantly different from baseline (pre‐exercise), *n* = 12. (b) Bland–Altman plot: differences between delta (Δ) pre to post CD4^+^ T cell counts between trial 1 and 2 by the mean of the two. Dashed lines indicate the upper and lower limits of the 95% confidence interval for the mean differences, *n* = 12.

The CD4^+^ T cell response to both 20/50 exercise trials was similar (*F*(1, 11) = 0.995, *P =* 0.340). There was a significant main effect of time (*F*(2, 22) = 25.544, *P* < 0.001) with acute increases in peripheral blood CD4^+^ from pre‐ ((0.86 ± 0.51) × 10^6^ cells/ml) to post‐exercise ((1.23 ± 0.55) × 10^6^ cells/ml) (*P* < 0.001), followed by a decrease towards baseline at 30‐min post‐exercise ((0.86 ± 0.52) × 10^6^ cells/ml) (*P* = 0.001). There was no significant interaction effect between trial and time point (*F*(2, 22) = 1.539, *P* = 0.237) (Figure 3a).

An excellent reliability in the response of CD4^+^ T cell counts to the 20/50 exercise trials was found with an ICC of 0.951 (95% CI: 0.814, 0.986). Bland–Altman analysis indicates that the differences in CD4^+^ T cell counts between trials ranged from 0.45 to −0.33 × 10^6^ cells/ml blood. One hundred percent of CD4^+^ T cell measurements lay within the upper (0.53 × 10^6^ cells/ml blood) and lower (−0.38 × 10^6^ cells/ml blood) limits of agreement (Figure 3b).

#### 3.2.3 CD8^+^ T cells

**FIGURE 4:**
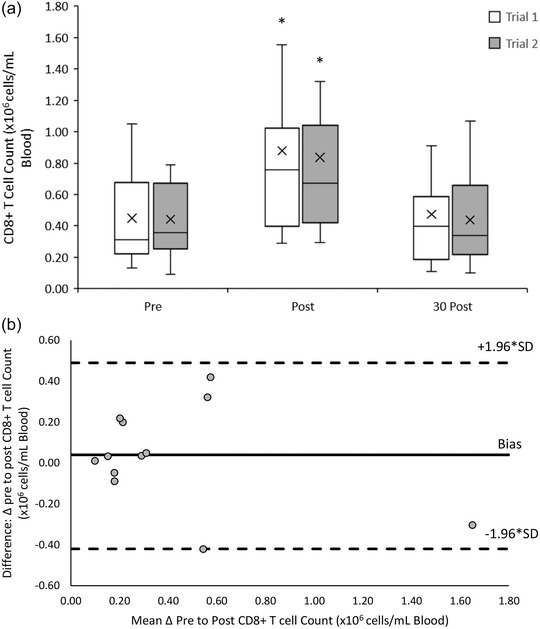
(a) CD8^+^ T cell counts pre‐, post‐ and 30 min post‐20/50 exercise bouts in both repeated trials. Counts are presented as means ± SD × 10^6^ cells/ml blood. *Means significantly different from baseline (pre‐exercise), *n* = 12. (b) Bland–Altman plot: differences between delta (Δ) pre to post CD8^+^ T cell counts between trial 1 and 2 by the mean of the two. Dashed lines indicate the upper and lower limits of the 95% confidence interval for the mean differences, *n* = 12.

The CD8^+^ T cell response to both 20/50 exercise trials was similar (*F*(1, 11) = 0.177, *P =* 0.682). There was a significant main effect of time (*F*(2, 22) = 45.123, *P* < 0.001) with acute increases in peripheral blood CD8^+^ from pre‐ ((0.45 ± 0.25) × 10^6^ cells/ml) to post‐exercise ((0.86 ± 0.61) × 10^6^ cells/ml) (*P* < 0.001), followed by a decrease towards baseline at 30 min post‐exercise ((0.46 ± 0.31) × 10^6^ cells/ml) (*P* < 0.001). There was no significant interaction effect between trial and time point (*F*(2, 22) = 0.496, *P* = 0.615) (Figure 4a).

An excellent reliability in the response of C8^+^ T cell counts to the 20/50 exercise trials was found with an ICC of 0.971 (95% CI: 0. 901, 0.991). Bland–Altman analysis indicates that the differences in CD8^+^ T cell counts between trials ranged from 0.42 to −0.42 × 10^6^ cells/ml blood. One hundred percent of CD8^+^ T cell measurements lay within the upper (0.49 × 10^6^ cells/ml blood) and lower (−0.42 × 10^6^ cells/ml blood) limits of agreement (Figure 4b).

### 3.3 Dendritic cells

#### 3.3.1 Total DCs (lineage‐ HLA‐DR^+^)

**FIGURE 5:**
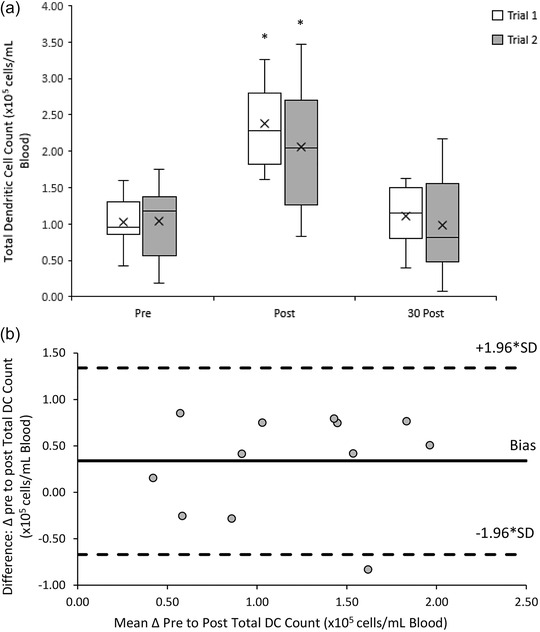
(a) Total DC counts pre‐, post‐ and 30 min post‐20/50 exercise bouts in both repeated trials. Counts are presented as means ± SD × 10^5^ cells/ml blood. *Means significantly different from baseline (pre‐exercise), *n* = 12. (b) Bland‐Altman plot: differences between delta (Δ) pre to post total DC counts between trial 1 and 2 by the mean of the two. Dashed lines indicate the upper and lower limits of the 95% confidence interval for the mean differences, *n* = 12.

The total‐DC response to both 20/50 exercise trials was similar (*F*(1, 11) = 1.254, *P =* 0.287). There was a significant main effect of time (*F*(2, 22) = 51.000, *P* < 0.001) with acute increases in peripheral blood total DCs from pre‐ ((1.03 ± 0.41) × 10^5^ cells/ml) to post‐exercise ((2.21 ± 0.73) × 10^5^ cells/ml) (*P* < 0.001), followed by a decrease towards baseline at 30 min post‐exercise ((1.05 ± 0.53) × 10^5^ cells/ml) (*P* < 0.001). There was no significant interaction effect between trial and time point (*F*(2, 22) = 3.596, *P* = 0.058) (Figure 5a).

A good reliability in the response of total‐DC counts to the 20/50 exercise trials was found with an ICC of 0.745 (95% CI: 0.189, 0.925). Bland–Altman analysis indicates that the differences in total‐DC counts between trials ranged from 0.85 to −0.83 × 10^5^ cells/ml blood. Ninety‐two percent (11 out of 12) of total‐DC measurements lay within the upper (1.34 × 10^5^ cells/ml blood) and lower (−0.67 × 10^5^ cells/ml blood) limits of agreement (Figure 5b).

#### 3.3.2 Myeloid DCs (CD11c^+^ CD123^−^)

**FIGURE 6:**
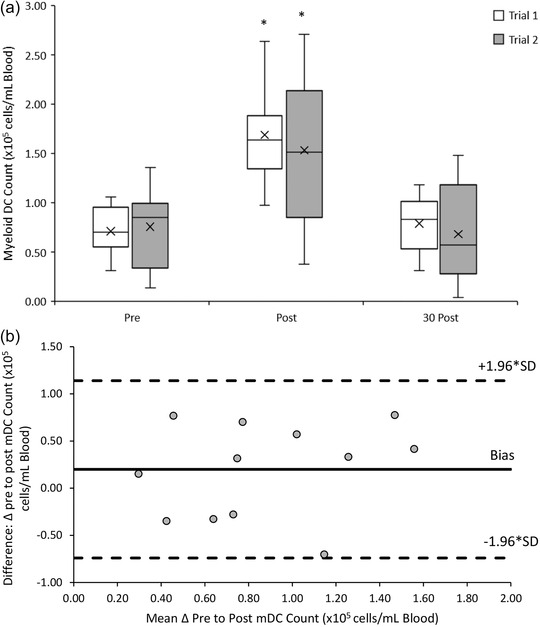
(a) Myeloid DC counts pre‐, post‐ and 30 min post‐20/50 exercise bouts in both repeated trials. Counts are presented as means ± SD × 10^5^ cells/ml blood. *Means significantly different to baseline (pre‐exercise), *n* = 12. (b) Bland–Altman plot: differences between delta (Δ) pre to post mDC counts between trial 1 and 2 by the mean of the two. Dashed lines indicate the upper and lower limits of the 95% confidence interval for the mean differences, *n* = 12.

The myeloid DC response to both 20/50 exercise trials was similar (*F*(1, 11) = 0.423, *P =* 0.529). There was a significant main effect of time (*F*(2, 22) = 41.233, *P* < 0.001) with acute increases in peripheral blood myeloid DCs from pre‐ ((0.74 ± 0.32) × 10^5^ cells/ml) to post‐exercise ((1.61 ± 0.61) × 10^5^ cells/ml) (*P* < 0.001), followed by a decrease towards baseline from at 30 min post‐exercise ((0.74 ± 0.39) × 10^5^ cells/ml) (*P* < 0.001). There was no significant interaction effect between trial and time point (*F*(2, 22) = 1.337, *P* = 0.282) (Figure 6a).

A good reliability in the response of myeloid DC counts to the 20/50 exercise trials was found with an ICC of 0.674 (95% CI: −0.111, 0.906). Bland–Altman analysis indicates that the differences in mDC counts between trials ranged from 0.78 to −0.70 × 10^5^ cells/ml blood. One hundred percent of mDC measurements lay within the upper (1.14 × 10^5^ cells/ml blood) and lower (−0.74 × 10^5^ cells/ml blood) limits of agreement (Figure 6b).

#### 3.3.3 Plasmacytoid DCs (CD11c^−^ CD123^+^)

**FIGURE 7:**
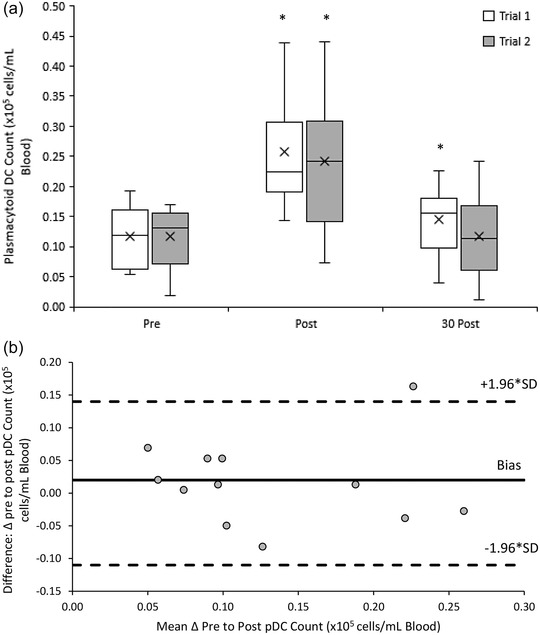
(a) Plasmacytoid DC counts pre‐, post‐ and 30 min post‐20/50 exercise bouts in both repeated trials. Counts are presented as means ± SD × 10^5^ cells/ml blood. *Means significantly different from baseline (pre‐exercise), *n* = 12. (b) Bland–Altman plot: differences between delta (Δ) pre to post pDC counts between trial 1 and 2 by the mean of the two. Dashed lines indicate the upper and lower limits of the 95% confidence interval for the mean differences, *n* = 12.

The pDC response to both 20/50 exercise trials was similar (*Z* = −1.225, *P* = 0.220). There was a significant main effect of time (χ^2^ (5) = 39.511, *P* < 0.001), with acute increases in peripheral blood pDCs from pre‐ ((0.12 ± 0.05) × 10^5^ cells/ml) to post‐exercise ((0.25 ± 0.11) × 10^5^ cells/ml) (*Z* = −3.059 *P* = 0.002) that remained elevated above baseline 30 min post‐exercise ((0.13 ± 0.06) × 10^5^ cells/ml) (*Z* = −2.510, *P* = 0.012) in trial 1. Acute increases in peripheral blood pDCs from pre‐ to post‐exercise (*Z* = −3.059, *P* = 0.002) were also found in trial 2 but decreased to baseline 30 min post‐exercise (*Z* = −0.628, *P* = 0.530) (Figure 7a).

An excellent reliability in the response of pDC counts to the 20/50 exercise trials was found with an ICC of 0.821 (95% CI: 0.384, 0.949). Bland–Altman analysis indicates that the differences in pDC counts between trials ranged from 0.16 to −0.08 × 10^5^ cells/ml blood. Ninety‐two percent (11 out of 12) of pDC measurements lay within the upper (0.14 × 10^5^ cells/ml blood) and lower (−0.11 × 10^5^ cells/ml blood) limits of agreement (Figure 7b).

## 4 DISCUSSION

This study aimed to establish the reproducibility of (1) immune cell counts, specifically T cells and DCs, and (2) physiological and perceived exertion responses to a 30‐min, high intensity cycle bout (20/50) to determine its usefulness as an exercise test to highlight immune alterations that may occur after periods of intensified training, or during NFOR/OTS. All T cell and DC numbers were elevated post‐exercise compared to pre‐exercise, and these differences did not differ between the repeated 20/50 trials. Therefore, the hypothesis that the 20/50 would induce robust and reproducible immune cell count changes can be accepted. It was also hypothesized that immune cell counts would increase during exercise from baseline and decline back to baseline 30 min post‐exercise. This can be accepted for total T cells (CD3^+^), CD4^+^ T cells, CD8^+^ T cells, total DCs and mDCs, but not for pDCs. pDCs remained elevated above baseline 30 min after the 20/50 bout in both trials. A secondary aim was to measure the reproducibility of the physiological (heart rate) and perceptual (ratings of perceived exertion) strain of the 20/50. Similar strains across both 20/50 exercise trials were found, which confirms that the physiological strain induced by the 20/50 may not be altered by repeated exposures. This is important if the 20/50 is to be used as a test to examine alterations of immune function during periods of heavy training stress.

Our findings showed robust increases in all T cell and DC counts from pre‐ to post‐ 20/50, followed by a reduction to baseline 30 min post‐20/50 in all T cells and DCs apart from pDCs. In line with our findings, previous research has also shown a biphasic response in T cells such that during exercise, a transient period of lymphocytosis occurs, followed by a period of lymphocytopenia after cessation of exercise (Shek et al., 1995), with a similar pattern of mobilization also shown in DCs (Brown et al., 2018; Ho et al., 2001). The increase in immune cells seen in our current study in the peripheral blood was likely to be driven by increased haemodynamics and the release of catecholamines following the activation of the sympathetic nervous system, and the decline driven largely by the release of glucocorticoids, such as cortisol via the HPA axis (Dimitrov et al., 2010; Hill et al., 2008; Krueger & Mooren, 2007). In agreement with this, the pattern of immune cell response to the 20/50 mirrors that of the cortisol response to the 55/80 stress test, whereby cortisol increased from pre‐ to post‐55/80, and decreased back to baseline 30 min post (Hough et al., 2021). This post‐exercise decline in immune cell numbers could represent a redistribution of effector cells for enhanced immune‐surveillance, and not exercise‐induced apoptosis (Peake et al., 2017; Simpson et al., 2020). The redistribution of immune cells with high effector functions is in line with the findings of the current study in which a preferential mobilization of CD8^+^ T cells occurred, increasing by 92% from pre‐ to post‐exercise compared to the 43% increase in CD4^+^ T helper cells.

The results of the current study indicate a 114% increase in total DCs to the 20/50, with a 113% and 119% increase in pDC and mDCs, respectively. All returned to baseline 30 min after cessation of the 20/50 apart from pDCs, which although they were on their way back down to baseline, were still significantly different to pre‐exercise pDC counts. Brown et al. (2018) showed that 20 min of cycling at 80% ${\dot{V}}_{O_{2}\max}$ increased total DC, pDC and mDC counts by 150%, 195% and 131%, respectively, which all returned to baseline 30 min after cessation of exercise. The slight differences in magnitude of DC count changes may be due to the intensity of exercise used. The 20‐min steady state cycle at 80% ${\dot{V}}_{O_{2}\max}$ used by Brown et al. (2018) elicited a mean HR of 176 ± 7 bpm and an RPE of 16 ± 1. These physiological and perceptual strains were higher than the mean HR and RPE across the two trials induced by our 20/50 (153 ± 15 bpm and 14 ± 1, respectively). It has been experimentally evidenced that the degree of DC mobilization is positively correlated with the concentration of catecholamine release into the blood during exercise, and that catecholamine release is increased with increasing exercise intensities (Suchanek et al., 2010; Zouhal et al., 2008). Therefore, differences in the magnitude of mobilization of DCs into the circulation could be due to differences in exercise intensity.

Additionally, previous groups have indicated a preferential mobilization of pDCs post‐exercise, compared to baseline (Brown et al., 2018; Suchanek et al., 2010). A vigorous ice‐hockey training session increased pDCs two‐fold compared to mDCs (200% vs. 100%, respectively) (Suchanek et al., 2010), and the 20‐min cycle at 80% ${\dot{V}}_{O_{2}\max}$ used by Brown et al. (2018) also induced a larger pDC mobilization than mDC (195% vs. 131%, respectively). This preferential mobilization of pDCs has been described as an adaptive process in which cells with potent anti‐viral properties are redistributed (Brown et al., 2018). pDCs have been shown to possess greater inflammatory and migratory potential compared to mDCs (Liu et al., 2021), so it may be unsurprising that previous studies have found a preferential mobilization of pDCs during exercise. As previously discussed, similarly to lymphocytes, the degree of DC mobilization during intense exercise is positively correlated with the concentration of catecholamines released into the blood (Suchanek et al., 2010), operating via a dose‐dependent increase in exercise intensity, and relying upon density of adrenergic receptors on DCs (Nijhuis et al., 2014). However, our findings did not show a preferential mobilization of pDCs. In fact, a slightly higher mDC mobilization than pDC (119% vs. 113%) was highlighted.

Our findings do, however, coincide with Deckx et al. (2015) whereby patients with multiple sclerosis and healthy controls undertook a moderate to high intensity mixed endurance (15 min cycle, 15 min walk) and resistance (3 × 10 repetitions; 6 upper and lower body exercise) exercise bout. They found a lower increase in pDCs (50%) compared to mDCs (75%), with no differences between patients and healthy controls. Of particular interest, in line with our findings, this study also found that pDCs took longer than 30 min to return to baseline, specifically they returned to baseline after 2 h of resting recovery. Differences in preferential mobilization could be due to the differences in exercise format, that is, the 20/50 was interval based and may not elicit the same DC response as a continuous exercise bout, or the exercise intensity elicited by the 20/50 may have induced a lower catecholamine response than Brown et al. (2018) and Suchanek et al. (2010). However, it is accepted that these hormones were not measured in the current study to confirm this argument.

Our analysis revealed that the reliability of T cell and DC count responses to the 20/50 can be interpreted as good (total DCs and mDCs) to excellent (total T cells, CD4^+^ T cells, CD8^+^ T cells and pDCs) according to ICC values, with no significant differences in cell count responses to exercise between the trials. All data points in the Bland–Altman analysis lay within the limits of agreement in 4 out of 6 cell types, with only one data point falling just outside for total DCs and pDCs, reinforcing conclusions drawn from the ICC values. The differences in the mean delta pre‐ to peak post‐exercise cell counts between trial 1 and 2 for all cell types are lower than their respective SRD measurements, meaning we can conclude that these differences are likely caused by measurement error, and not systematic variability between trials (Vaz et al., 2013). These reliability results indicate that if implemented before and after a period of intensified training, any changes seen in the responses to the 20/50 are likely to represent actual immune alterations associated with negative states of overtraining and are not due to regular variation. As previously stated, there are currently no clear reliable biomarkers of overtraining capable of identifying the occurrence of NFOR/OTS. The results of the current study suggest that the 20/50 is a test capable of identifying whether immune alterations could be a useful biomarker in highlighting the occurrence of NFOR/OTS.

The ICC is commonly used as a measure of relative reliability, as it can conveniently categorise the reliability of a measure as poor, moderate, good or excellent (Hartmann et al., 2023; Koo & Li, 2016). However, caution must be taken when utilizing ICC as a sole measure of reliability, as the high within‐group standard deviation associated with a very heterogeneous population may lead to a high ICC value regardless of how unreliable the method is (Hartmann et al., 2023). As such, it is argued that the ICC does not provide a comprehensive assessment of reliability when taken alone. Calculating the coefficient of variation as another measure of relative reliability is therefore seen as beneficial for interpretation (Gomez & Gomez, 1984). Nevertheless, the CV is also not without its flaws, as when the mean is close to zero, inaccurate results can occur, which is likely the cause of the inflated CV values in the current study (Hartmann et al., 2023). As such, the addition of absolute reliability measurements via the Bland–Altman plot and calculation of the smallest real difference is required to provide a more comprehensive review of overall reliability. Bland–Altman analysis provides a visual interpretation of measurement agreements whereby a reference range within which 95% of all differences between measurements are likely to lie (Mansournia et al., 2021). The smallest real difference is easily interpretable because it provides an estimate of the maximal difference there will be between two measurements on 95% of occasions and is particularly important for clinical translation as it is reported in the same units as the measurement itself (Vaz et al., 2013). Vitally, our calculation of SRD took the *n* into account, rather than the more widely used 1.96 × SEM × √2.

To conclude, the 20/50 exercise test elicited robust and reproducible DC and T cell responses. Consequently, when implemented before and after a period of intensified training, the 20/50 exercise test is capable of identifying whether immune alterations are a useful biomarker in highlighting the occurrence of NFOR/OTS. The development of this test is important when considering the high incidence of NFOR/OTS in the athletic population. However, a limitation of the current work is that all participants were male. Females could potentially display a different immune response to the same stimulus, and therefore in order to make a viable test, both biological sexes should be assessed. Future work should investigate exercise induced immunological changes relating to DCs and T cells associated with intensified training by implementing the 20/50 before and after a period of intensive training. This would provide an overview of how the relationship between the innate and adaptive systems may be altered with intensified training, and/or when in a negative state of overtraining.

## AUTHOR CONTRIBUTIONS

Carla Baker, Jessica Piasecki, John A. Hunt and John Hough were responsible for the conception or design of the work, acquiring, analysing and interpreting the data and critically revising the work for important intellectual content. All authors have read and approved the final version of this manuscript and agree to be accountable for all aspects of the work in ensuring that questions related to the accuracy or integrity of any part of the work are appropriately investigated and resolved. All persons designated as authors qualify for authorship, and all those who qualify for authorship are listed.

## CONFLICT OF INTEREST

The authors declare no conflicts of interest.

## FUNDING INFORMATION

No funding was received for this work.

## Data Availability

Data are available as supporting information. Flow cytometry outputs are available from the authors on request.
